# Chronic myelogenous leukaemia exosomes modulate bone marrow microenvironment through activation of epidermal growth factor receptor

**DOI:** 10.1111/jcmm.12873

**Published:** 2016-05-14

**Authors:** Chiara Corrado, Laura Saieva, Stefania Raimondo, Alessandra Santoro, Giacomo De Leo, Riccardo Alessandro

**Affiliations:** ^1^ Dipartimento di Biopatologia e Biotecnologie MedicheUniversità degli studi di Palermosezione di Biologia e GeneticaPalermoItaly; ^2^ Divisione di EmatologiaA.O. Ospedali Riuniti Villa Sofia‐CervelloPalermoItaly

**Keywords:** exosomes, chronic myeloid leukaemia, epidermal growth factor receptor, SNAIL, interleukin 8

## Abstract

Chronic myelogenous leukaemia (CML) is a clonal myeloproliferative disorder. Recent evidence indicates that altered crosstalk between CML and mesenchymal stromal cells may affect leukaemia survival; moreover, vesicles released by both tumour and non‐tumour cells into the microenvironment provide a suitable niche for cancer cell growth and survival. We previously demonstrated that leukaemic and stromal cells establish an exosome‐mediated bidirectional crosstalk leading to the production of IL8 in stromal cells, thus sustaining the survival of CML cells. Human cell lines used are LAMA84 (CML cells), HS5 (stromal cells) and bone marrow primary stromal cells; gene expression and protein analysis were performed by real‐time PCR and Western blot. IL8 and MMP9 secretions were evaluated by ELISA. Exosomes were isolated from CML cells and blood samples of CML patients. Here, we show that LAMA84 and CML patients’ exosomes contain amphiregulin (AREG), thus activating epidermal growth factor receptor (EGFR) signalling in stromal cells. EGFR signalling increases the expression of SNAIL and its targets, MMP9 and IL8. We also demonstrated that pre‐treatment of HS5 with LAMA84 exosomes increases the expression of annexin A2 that promotes the adhesion of leukaemic cells to the stromal monolayer, finally supporting the growth and invasiveness of leukaemic cells. Leukaemic and stromal cells establish a bidirectional crosstalk: exosomes promote proliferation and survival of leukaemic cells, both *in vitro* and *in vivo*, by inducing IL8 secretion from stromal cells. We propose that this mechanism is activated by a ligand–receptor interaction between AREG, found in CML exosomes, and EGFR in bone marrow stromal cells.

## Introduction

Chronic myelogenous leukaemia (CML) is a clonal myeloproliferative disorder characterized by the reciprocal t (9:22) chromosomal translocation that results in the Philadelphia (Ph) chromosome encoding the chimeric Bcr–Abl oncoprotein 1. Bcr–Abl shows a constitutive tyrosine kinase activity that drives disease progression by stimulating a number of downstream signalling pathways 2. Because of its critical role in CML pathogenesis, most therapies have focused on targeting Bcr–Abl 3. These conventional therapies, such as imatinib or second‐ and third‐generation inhibitors (dasatinib or nilotinib), induce molecular remission in most patients; however, a number of investigations have demonstrated the persistence of a leukaemic stem cell pool in the bone marrow (BM) niche even after imatinib treatment 4. Recent evidence indicates that altered crosstalk between mesenchymal stromal cells and CML cells may affect leukaemia cell survival and resistance to chemotherapy 5, 6. For these reasons, new studies have shifted the focus on the role of cytokines, growth factors, adhesion molecules released by both tumour and non‐tumour cells into the microenvironment that provide a suitable niche for cancer cell growth and survival. In this context, exosomes are now considered as new actors in regulating tumour microenvironment 7, 8.

Exosomes are nanovesicles of 40–100 nm diameter that are formed within the endosomal compartment and secreted when a multivesicular body (MVB) fuses with the plasma membrane 9. We have recently shown that cancer‐derived exosomes modulate the crosstalk between leukaemia cells and the bone marrow microenvironment; in particular, we have reported that CML cells release exosomes and that the addition of these vesicles to vascular endothelial cells as well as to bone marrow stromal cells affects both *in vitro* and *in vivo* tumour progression, through the stimulation of an interleukin 8‐mediated paracrine and autocrine loops 10, 11, 12, 13.

Different studies suggest that epidermal growth factor receptor (EGFR) ligands regulate autocrine and/or paracrine signalling inducing the activation of EGFR targets such as early‐growth response‐1 that drives stromal cells to produce several cytokines or growth factors that regulate cell proliferation, migration and apoptosis 14, 15. Moreover, EGFR ligands, such as amphiregulin (AREG), are able to activate EGFR, in a paracrine or autocrine way, thus enhancing tumour cell aggressiveness and chemoresistance and contributing to the transformed phenotype 16, 17, 18. Singh and Coffey recently proposed the extracrine (exosomal‐targeted receptor activation) signalling that involves the packaging and release of EGFR ligands *via* exosomes 19. Coffey's group demonstrated that tumour exosomes, carrying the EGFR ligand AREG, are rapidly internalized in recipient human breast and colorectal cancer cells thus increasing cancer cell invasion 20. AREG can be considered a multicrine signalling protein, involved in evading apoptosis and sustaining angiogenesis, tissue invasion and metastasis 17, 21, 22. Here, we show that CML exosomes, carrying AREG, are able to activate EGFR signalling in stromal cells leading to increased IL8 expression and secretion 12.

Annexin A2 is a pleiotropic protein involved in the regulation of different cellular processes, such as cellular growth, cell adhesion and motility. Recently, it has been demonstrated that annexin A2, expressed on stromal cells, regulated bone marrow homing of Multiple Myeloma cells supporting their growth and regulating their adhesion to stromal cells 23. We demonstrated that the pre‐treatment of HS5 cells with LAMA84 exosomes increases the expression of annexin A2 mRNA and protein and the adhesion of leukaemic cells to the stromal monolayer, thus sustaining the growth, survival and invasiveness of CML cells 12.

In conclusion, we showed that chronic myelogenous leukaemia cell‐derived exosomes modulate bone marrow microenvironment through activation of EGFR in stromal cells. These results may have significant implications for new therapeutic approaches involving exosomes and their specific content for early diagnosis of chronic myelogenous leukaemia.

## Materials and methods

### Cell culture and reagents

Chronic myeloid leukaemia cell line, LAMA84, was obtained from DSMZ (Braunschweig, Germany); human primary CD34^+^ cells and bone marrow primary stromal cells (BMSCs) were obtained from Lonza (Basel, Switzerland). LAMA84 cells were cultured in RPMI 1640 medium, supplemented with 10% foetal bovine serum (FBS), 2 mM L‐glutamine, 100 U/ml penicillin and 100 μg/ml streptomycin (Euroclone, UK). CD34^+^ cells were cultured in IMDM medium, supplemented with 15% FBS, 2 mM L‐glutamine, 100 U/ml penicillin and 100 mg/ml streptomycin (Euroclone, UK). BMSCs were cultured in MyeloCult H5100 (STEMCELL Technologies Inc., Vancouver, BC, Canada). Gefitinib or Erlotinib (Cayman Chemical, Ann Arbor, MI, USA) was solubilized at 10‐mM stock solution in DMSO and stored at −20°C. Neutralizing antibody anti‐AREG (R&D Systems, Abingdon, UK) was reconstituted at 0.2 mg/ml in sterile PBS, aliquoted and stored at −20°C. Recombinant Areg (R&D Systems, Abingdon, UK) was reconstituted at 0.1 mg/ml in sterile PBS, aliquoted and stored at −20°C. Working dilutions, where necessary, were prepared in medium. All other reagents were purchased from Sigma‐Aldrich (St. Louis, MO, USA), if not cited otherwise.

### CML patients

Blood samples were obtained from 13 newly diagnosed CML patients. Informed consent was obtained from patients, according to the Declaration of Helsinki and with hospital ethics committee approval. Whole‐blood samples were treated with red blood cell lysing buffer (Sigma, St. Louis, MO, USA) for 2 min at room temperature and then centrifuged at 350 g for 7 min to recover and discard lysed red cells. The interphase layer containing CML cells was collected, and the cells were resuspended in PBS and used to obtain total protein extract and to extract RNA or cultured in RPMI complete medium as described for LAMA84 cells. Exosomes released in fresh patient's serum were prepared as described in the section ‘Exosome isolation’.

### Exosome isolation

Exosomes released by CML cells after a 24‐hr culture period in the presence of FBS previously ultracentrifuged (vesicle‐free media) were isolated from conditioned culture medium by differential centrifugation as described by Thiery *et al*. 24. Exosome pellet was washed and then resuspended in PBS. Exosome protein content was determined by the Bradford assay (Pierce, Rockford, IL, USA). Exosome characterization was previously described by our group 11.

### RNA extraction and real‐time PCR

HS5 cells were grown in 12‐well plates up to 80% confluence and treated or not with 20–50 μg/ml of LAMA84− exosomes ± Gefitinib 30 μM for different times (30 min. to 72 h), as described in the results. Bone marrow primary stromal cells were grown in 12‐well plates up to 80% confluence and treated or not with 50 μg/ml of LAMA84− exosomes ± Gefitinib 30 μM for different times (1–6 h), as described in the results. RNA was extracted using the commercially available Illustra RNAspin Mini Isolation Kit (GE Healthcare, Little Chalfont, Buckinghamshire, UK), according to manufacturer's instructions. Total RNA from HS5 or from CML patient cells was reverse transcribed to cDNA using the High Capacity cDNA Reverse Transcription Kit (Applied Biosystems, Foster City, CA, USA). RT‐QPCR was performed in 48‐well plates using the Step‐One Real‐Time PCR system (Applied Biosystems). For quantitative Sybergreen real‐time PCR, reaction was carried out in a total volume of 20 μl containing 2× SYBR Green I Master Mix (Applied Biosystems), 2 μl cDNA and 300 nM forward and reverse primers. Primer sequences were as follows:

GAPDH (5′ATGGGGAAGGTGAAGGTCG3′, 5′GGGTCATTGATGGCAACAATAT3′)
SNAIL (5′GCGAGCTGCAGGACTCTAAT3′, 5′CCCGCAATGGTCCACAAAAC3′)
IL8 (5′GAATGGGTTTGCTAGAATGTGATA3′, 5′CAGACTAGGGTTGCCAGATTTAAC3′)
MMP9 (5′CGCTACCACCTCGAACTTTG3′, 5′GCCATTCACGTCGTCCTTAT3′)
ANNEXIN A2 (5′TGAGCGGGATGCTTTGAAC3′¸ 5′ATCCTGTCTCTGTGCATTGCTG3′)
AREG (5′GTGGTGCTGTCGCTCTTGATACTC3′, 5′TCAAATCCATCAGCACTGTGGTC3′)
HBEGF (5′GGAACTCACTTTCCCTTGTGTC3′, 5′CTCAGCCTTTTGCTTTGCTAAT3′)
EREG (5′GAGAAGGGGGAGTAATGACTTG3′, 5′AAGTGCAATTACAGAGTGCAAAA3′)
EGF (5′TGCAGAGGGATACGCCCTAA3′, 5′TGCGTGGACAGGAAACAAGT3′)

All were obtained from Invitrogen (Foster City, CA, USA). Real‐time PCR was performed in duplicates for each data point. Relative changes in gene expression between control and treated samples were determined using the ΔΔCt method. Levels of the target transcript were normalized to a GAPDH endogenous control, constantly expressed in all samples (ΔCt). For ΔΔCt values, additional subtractions were performed between treated samples and control ΔCt values. Final values were expressed as fold of induction.

### RNA interference

Small‐interfering RNAs (siRNA) targeting Snail or scramble siRNA were purchased from Dharmacon (ON‐TARGET plus SMART pool, Human, Dharmacon Inc., Lafayette, CO, USA) and used to transfect HS5 cells by employing Lipofectamine RNAiMAX Reagent (Invitrogen, UK), according to the suggested protocol. Briefly, 30–50% confluent cells, plated the day before in 24 wells, were incubated with a mix of Lipofectamine RNAiMAX‐Opti‐MEM containing 2 pmol of siRNA. After 6 h, cells were lysed for RNA extraction. Knockdown efficiency was determined by real‐time PCR quantitation of Snail.

### Western blot

HS5 cells were starved for 2 h in a serum‐free medium and treated or not for different times (30 min., 3 h, 6 h, 24 h) with LAMA84− exosomes (20–50 μg/ml) ± gefitinib 30 μM or erlotinib 30 μM. Neutralizing antibody anti‐AREG (20 ng/ml) was incubated with LAMA84 exosomes (50 μg/ml) for 2 h at 37°C and then used to treat HS5 for 3 h. Bone marrow primary stromal cells were treated or not for different times (30 min., 18 h) with LAMA84− exosomes (50 μg/ml) ± gefitinib 30 μM. Total protein lysates were obtained at the end of each treatment. Protein lysates from LAMA84 cells, from CML patients cells, from HS5, from BMSC, from exosomes or from conditioned medium of LAMA84 cells were analysed by SDS‐PAGE in reducing conditions followed by Western blotting as previously described 6. Antibodies used in the experiments were as follows: anti‐EGFR, pEGFR, annexin A2 and β‐actin (all from Cell Signalling Technology, Lane Danvers, MA, USA); anti‐SNAIL and MMP9 (all from Santa Cruz Biotechnology, Santa Cruz, CA, USA) and anti‐AREG (R&D systems, Abingdon, UK).

### ELISA assay

HS5‐conditioned medium (CM) was collected from cells stimulated or not for 12–24 h with LAMA84 exosomes (20–50 μg/ml) ± 30 μM gefitinib. BMSC‐CM was collected from cells stimulated or not for 6 h with LAMA84− exosomes (50 μg/ml) ± 30 μM gefitinib. CM aliquots were centrifuged to remove cellular debris and used to quantify IL8 or MMP9 with ELISA kits according to the manufacturer's protocol (R&D Systems, Abingdon, UK, for IL8; Cloud‐Clone Corp., Houston, TX, USA, for MMP9).

### Adhesion assay

Adhesion assay was performed as previously described by our group 12. Briefly, HS5 monolayer was incubated for 24 h with 50 μg/ml LAMA84− exosomes ± gefitinib (1–5–10 μM). After treatments, cells were washed with PBS, fixed and CML cells were added for 3.5 h at 37°C. Adherent cells were stained with haematoxylin/eosin, each test group was assayed in triplicate; five high power (400×) fields were counted for each condition.

### Statistical analysis

Data were expressed as mean ± SEMs of three independent experiments. Statistical analysis was performed by using an unpaired Student's *t*‐test. Differences were considered to be significant when *P* values were smaller than 0.05.

## Results

### LAMA84 cells and CML patients release exosomes containing AREG

**Figure 1:**
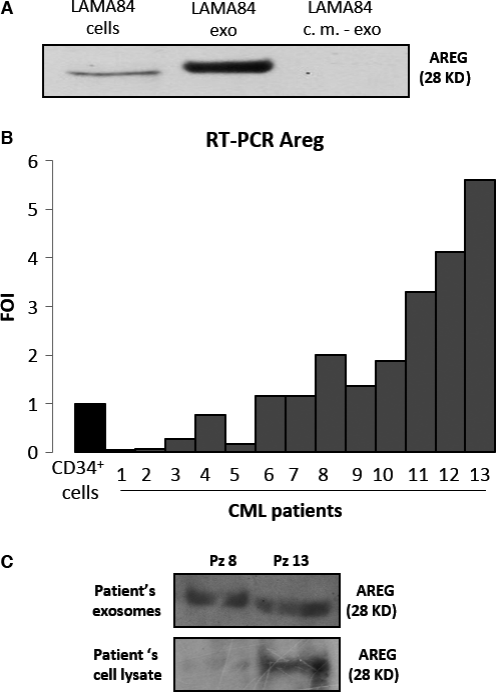
Exosomes isolated from LAMA84 cells and CML patient cells contain amphiregulin. (**A**) Total proteins were extracted from LAMA84 cells, LAMA84− exosomes (exo) and conditioned medium of cells deprived of exo (c.m.–exo) and were subjected to Western blot analysis with antibody against AREG. LAMA84− exosomes are enriched with epidermal growth factor receptor (EGFR) ligand, AREG. (**B**) Analysis, by real‐time PCR, of AREG mRNA levels from CD34^+^ cells, as control, and from cells of 13 different CML patients. Sixty‐two per cent of these patients showed an increase in AREG mRNA expression. Levels of the target transcript were normalized to a GAPDH endogenous control. Final values were expressed as fold of induction. (**C**) Total proteins were extracted from cells of CML patients n°8 and n°13 and from exosomes isolated from serum of the same CML patients and were subjected to Western blot analysis with antibody against AREG. The protein is found both in lysates from cell patients (patient's cell lysate) and in lysates from exosomes isolated from patient's serum (patient's exosomes). AREG, amphiregulin; CML, chronic myelogenous leukaemia.

In order to better understand how the addition of LAMA84− exosomes to bone marrow stromal cells leads to the increased interleukin 8 release 12, we decided to investigate the possible role of receptor tyrosine kinases. We have observed, by RTK assay, that the treatment of HS5 stromal cells with LAMA84− exosomes for 30 min. induces the activation of EGFR (Fig. S1). We, therefore, investigated the expression of EGFR ligands in LAMA84 cells, CML patients and in their released exosomes. We determined the mRNA expression of different EGFR ligands on LAMA84 cells, and we found that the most expressed EGFR ligand is AREG (Fig. S2C). Recent articles correlate AREG to tumour cell aggressiveness and chemoresistance 16, 17, 18, 21, 22, and it has been proposed a new extracrine (exosomal‐targeted receptor activation) signalling that involves the packaging and release of the EGFR ligand AREG *via* exosomes 20. LAMA84 cells and their exosomes contain Areg mRNA, but no significative differences between cells and exosomes were found (data not shown). As shown, by Western blot, in Figure 1A, AREG is enriched in LAMA84− exosomes with respect to LAMA84 cells. Moreover, the conditioned medium of LAMA84 cells deprived of exosomes does not contain the protein, thus confirming the localization of AREG in exosomes. Relatively to protein content of other EGFR ligands on LAMA84 cells and LAMA84− exosomes, we performed the luminex assay and we found that transforming growth factor alpha (TGF‐α) is undetermined both in cells and in exosomes, while EGF and HBEGF are found in very small amount with a detection fold closed to the lower standard, both in cells and in exosomes (data not shown). To confirm the presence of EGFR ligands also *in vivo,* we collected blood samples from 13 newly diagnosed CML patients and extracted RNAs; the real‐time PCR showed that CML patients expressed mRNA of HBEGF, EREG and AREG (Fig. 1B and Fig. S2A and B). Figure 1B shows, by real‐time PCR, that all patients express Areg mRNA;, moreover, eight patients (62%) showed higher level of Areg mRNA with respect to CD34^+^ control cells. To confirm *in vivo* the packaging of AREG in exosomes, we collected cells and exosomes from two patients to obtain total protein lysate. As shown in Figure 1C, both patients contain the ligand in the cells and in the exosomes. These data demonstrate the presence of AREG in CML cells *in vitro* and *in vivo*.

### LAMA84− exosomes increase EGFR phosphorylation in HS5 stromal cells and in bone marrow primary stromal cells

**Figure 2:**
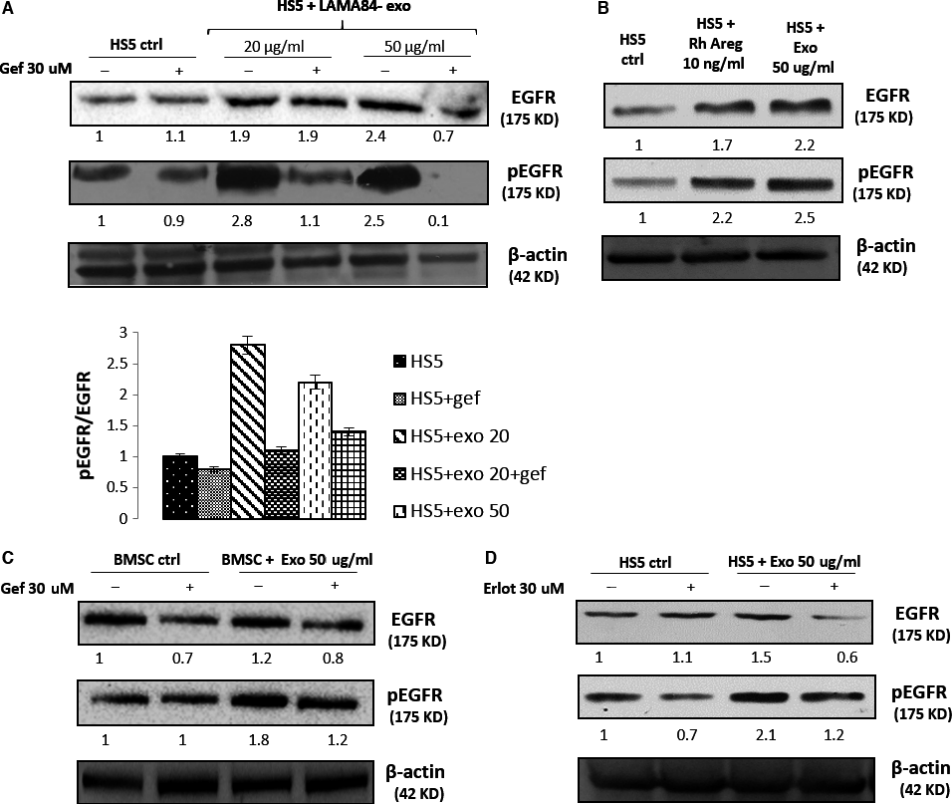
LAMA84− exosomes increase EGFR phosphorylation in HS5 stromal cells and in bone marrow primary stromal cells. Total proteins were extracted from HS5 cells or BMSCs treated for 30 min. with different conditions were subjected to Western blot analysis with antibody against EGFR and pEGFR. The numbers represent the values of densitometric analysis of EGFR and pEGFR levels, normalized *versus* β‐actin, used as loading control. The histogram below represents the ratio pEGFR/EGFR, based on densitometric analysis. Results from three independent experiments are shown. (**A**) HS5 treated or not with LAMA84− exosomes (20–50 μg/ml) ± 30 μM gefitinib. (**B**) HS5 treated or not with recombinant Areg (Rh Areg 10 ng/ml) or LAMA84− exosomes (50 μg/ml). (**C**) BMSC treated or not with LAMA84− exosomes (50 μg/ml) ± 30 μM gefitinib. (**D**) HS5 treated or not with LAMA84− exosomes (50 μg/ml) ± 30 μM erlotinib. AREG, amphiregulin; BMSC, bone marrow primary stromal cells; EGFR, epidermal growth factor receptor.

In order to confirm the functional role of exosomal AREG as signalling molecule in stromal cells, HS5 cells were treated for 30 min. with different concentrations of LAMA84− exosomes (20–50 μg/ml) and Gefitinib, the selective inhibitor of the EGFR activation. Figure 2A and B shows, by Western blot, that LAMA84− exosomes increase the expression of EGFR and its phosphorylation at levels comparable with the effect of recombinant AREG, used as positive control. Co‐treatment with LAMA84− exosomes and gefitinib significantly reduces the phosphorylation of EGFR. These data suggest that LAMA84− exosomes trigger an AREG‐mediated activation of EGFR in HS5 stromal cells. These data were also confirmed using bone marrow primary stromal cells (Fig. 2C). To further endorse the effect of LAMA84− exosomes on EGFR phosphorylation, we performed a Western blot assay on protein extracts from HS5 cells treated or not with LAMA84− exosomes (50 μg/ml) and 30 μM erlotinib, another inhibitor of the EGFR activation. As shown in Figure 2D, LAMA84− exosomes increase the EGFR phosphorylation and erlotinib partially reverts these effects.

### LAMA84− exosomes increase Snail expression in HS5 stromal cells and in bone marrow primary stromal cells

**Figure 3:**
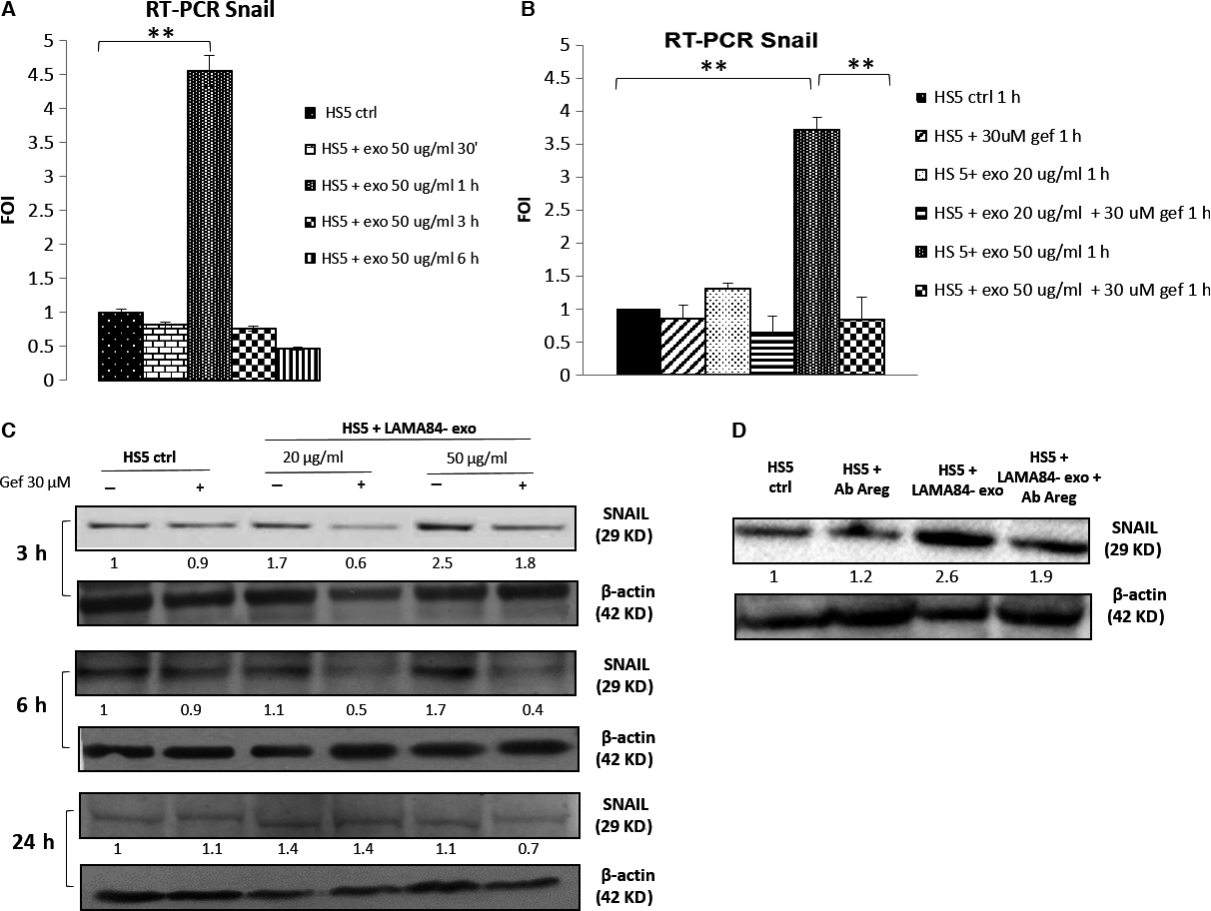
LAMA84− exosomes increase Snail expression in HS5 stromal cells. Analysis, by real‐time PCR, of Snail mRNA levels from HS5 treated with 50 μg/ml of LAMA84− exosomes for different times (**A**) or HS5 treated for 1 h with LAMA84− exosomes (20–50 μg/ml) ± 30 μM gefitinib (**B**). LAMA84− exosomes induce a significative increase in Snail at 1 h, and gefitinib reverts this effect. Levels of the target transcript were normalized to GAPDH as endogenous control. Final values were expressed as fold of induction. Results from three independent experiments are shown. **P < 0.01. Total proteins were extracted from HS5 cells and HS5 treated for 3–6–24 h with LAMA84− exosomes (20–50 μg/ml) ± 30 μM gefitinib (**C**) and LAMA84− exosomes plus neutralizing antibody anti‐AREG (previously incubated for 2 h at 37°C; (**D**) and were subjected to Western blot analysis with antibody against SNAIL. LAMA84− exosomes induce an increase in SNAIL in a dose‐dependent manner; co‐treatment of LAMA84− exosomes and Ab AREG partially reverts this effect confirming the involvement of AREG. The numbers represent the values of densitometric analysis of SNAIL, normalized *versus* β‐actin, used as loading control. Results from three independent experiments are shown. AREG, amphiregulin.

To further investigate the involvement of EGFR signalling pathway in exosome‐mediated events, we measured the expression level of SNAIL, a downstream target of EGFR 25, 26. For this purpose, HS5 cells were treated for different times (up to 24 h) with 50 μg/ml of LAMA84− exosomes. As shown in Figure 3A, LAMA84− exosomes induce a significative increase in mRNA expression of Snail after 1 h of treatment. Treatment of HS5 cells with increasing concentration of LAMA84− exosomes (20–50 μg/ml) confirms that 50 μg/ml of exosomes causes the maximum effect. Furthermore, the co‐treatment of LAMA84− exosomes with gefitinib significantly reduces the expression of Snail, at mRNA level, thus reverting the effects mediated by LAMA84− exosomes and confirming the involvement of EGFR‐mediated pathway (Fig. 3B). These results were also confirmed at protein level, as shown by Western blot in Figure 3C. HS5 cells were treated up to 24 h with 20 or 50 μg/ml of LAMA84− exosomes and with LAMA84− exosomes plus gefitinib; LAMA84− exosomes increase the expression of SNAIL with the maximum effect at 3 h and gefitinib reverts the effects observed. Data were confirmed by treatment of HS5 stromal cells for 3 h with neutralizing antibody anti‐AREG (Ab Areg) in combination or not with LAMA84− exosomes (50 μg/ml). Figure 3D shows that the treatment of cells with Ab Areg reduces the exosomal‐induced expression of Snail. These data demonstrate that (i) LAMA84− exosomes increase Snail expression both at mRNA and at protein level in HS5 stromal cells and (ii) gefitinib, as well as the neutralizing antibody to AREG, reverts the effects mediated by exosomes. Moreover, we demonstrated that LAMA84− exosomes exert the same effects on bone marrow primary stromal cells, as shown in Figure S3B and E.

### LAMA84− exosomes increase the expression of IL8 and MMP9 in HS5 stromal cells and in bone marrow primary stromal cells

**Figure 4:**
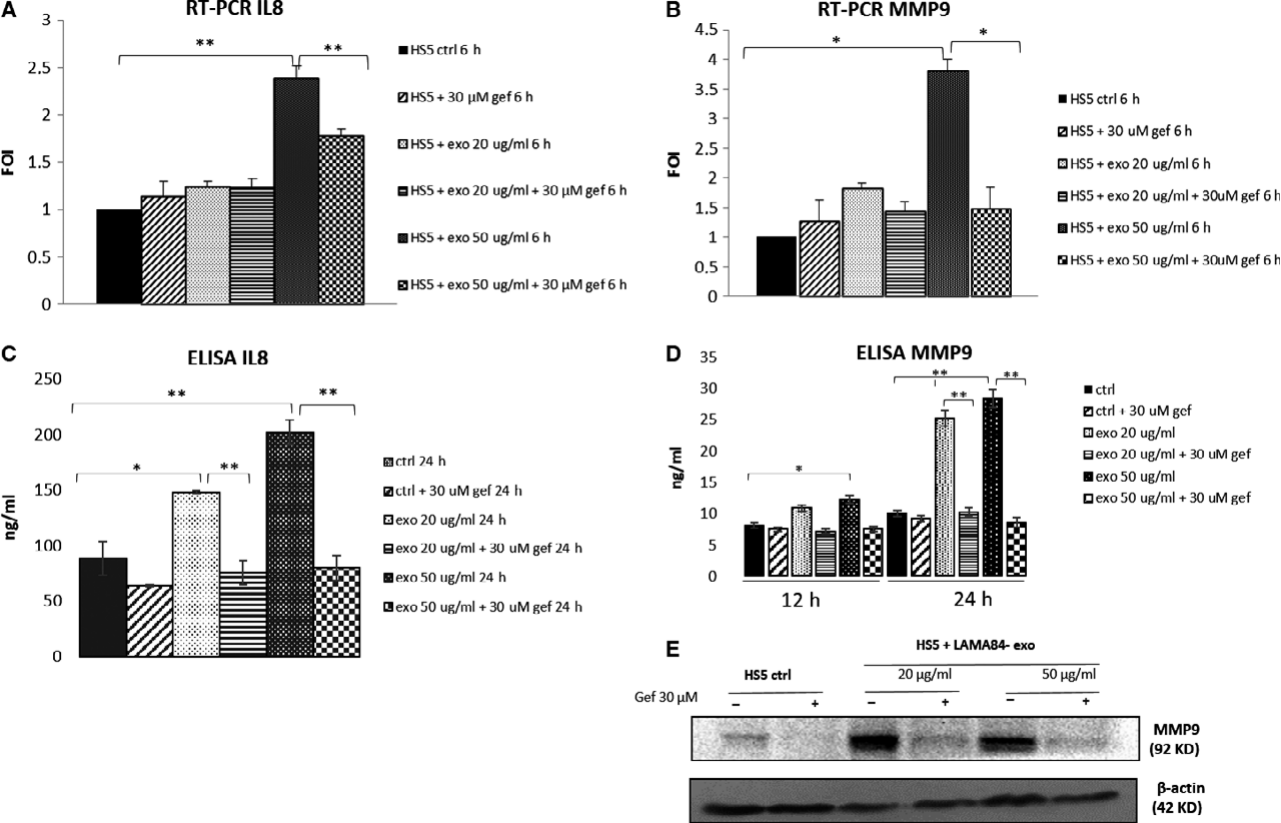
LAMA84− exosomes increase the expression of IL8 and MMP9 in HS5 stromal cells. Analysis, by real‐time PCR, of IL8 (**A**) and MMP9 (**B**) mRNA expression from HS5 treated for 6 h with LAMA84− exosomes (20–50 μg/ml) ± 30 μM gefitinib. Levels of the target transcript were normalized to GAPDH as endogenous control. Final values were expressed as fold of induction. The conditioned medium of HS5, treated for 12–24 h with LAMA84− exosomes, was used for IL8 ELISA (**C**) or MMP9 ELISA (**D**). Total proteins were extracted from HS5 cells and HS5 treated for 24 h with LAMA84− exosomes (20–50 μg/ml) ± 30 μM gefitinib and were subjected to Western blot analysis with antibody against MMP9; β‐actin was used as loading control (**E**). LAMA84− exosomes induce a significative increase in IL8 and MMP9 (mRNA and protein) and gefitinib reverts this effects. Results from three independent experiments are shown. *P < 0.05; **P < 0.01.

**Figure 5:**
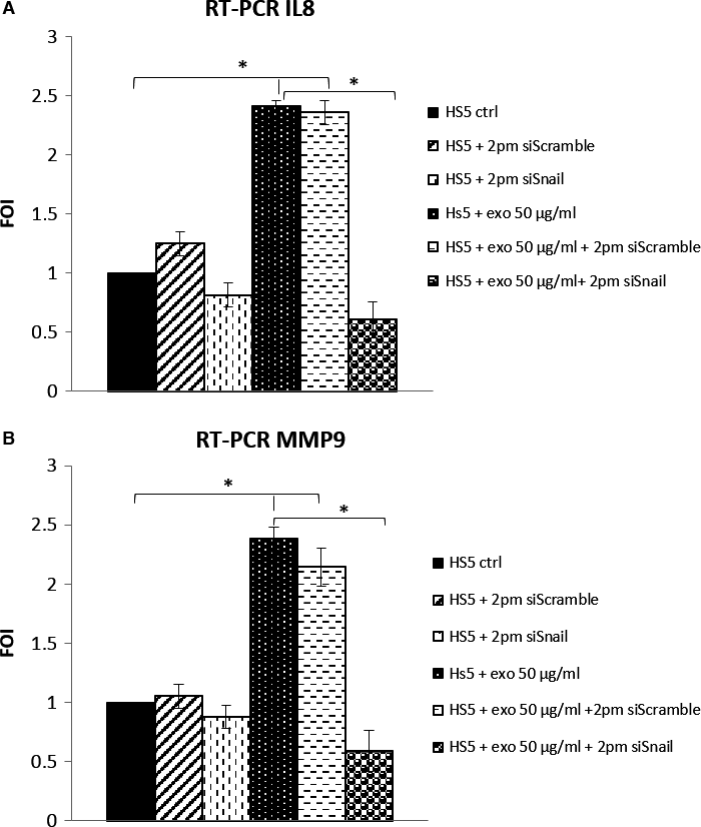
SNAIL siRNA inhibits IL8 and MMP9 mRNA expression from HS5 cells. IL8(**A**) and MMP9 (**B**) mRNA expression levels were evaluated in HS5 transfected with scramble siRNA (siScramble) or with Snail siRNA (siSnail). Transfection of HS5 with Snail siRNA reduced IL8 and MMP9 mRNA levels compared with control (ctrl). Levels of the target transcript were normalized to GAPDH as endogenous control. Final values were expressed as fold of induction. Results from three independent experiments are shown. *P < 0.05.

We demonstrated that LAMA84− exosomes carrying AREG activate EGFR on stromal cells, thus inducing the downstream signalling and in particular the expression of SNAIL (Fig. 3). IL8 and MMP9 are two known genes that are regulated by Snail. Figure 4A and B shows, by real‐time PCR at 6 h, that the treatment of HS5 cells with LAMA84− exosomes induces a significative dose‐dependent increase in IL8 (Fig. 4A) and MMP9 (Fig. 4B) expression; data were confirmed at protein level with a dose‐dependent increase in IL8 and MMP9 at 24 h, demonstrated by ELISA (IL8 and MMP9, Fig. 4C and D) and by Western blot (MMP9, Fig. 4E). To confirm the involvement of EGFR signalling, addition of gefitinib to HS5 cells reverts the effects observed both at mRNA and at protein level. Moreover, we demonstrated that LAMA84− exosomes exert the same effects on bone marrow primary stromal cells, as shown in Figure S3A, C, D and E. In conclusion, we showed here that the increase in the expression of IL8 and MMP9, induced by addition of LAMA84− exosomes, is mediated by EGFR pathway. To confirm the essential role of Snail in EGFR signalling, we also used small‐interfering RNAs (siRNAs) to knock down Snail expression in HS5 cells. Transfection of HS5 with Snail siRNA caused, as expected, a striking reduction in both IL8 (Fig. 5A) and MMP9 (Fig. 5B) mRNA levels compared with HS5 transfected with scramble siRNA. These results confirmed the central role of Snail in regulating IL8 and MMP9 expression.

### Treatment of HS5 with LAMA84− exosomes increases LAMA84 cell adhesion to stromal monolayer through the expression of annexin A2

**Figure 6:**
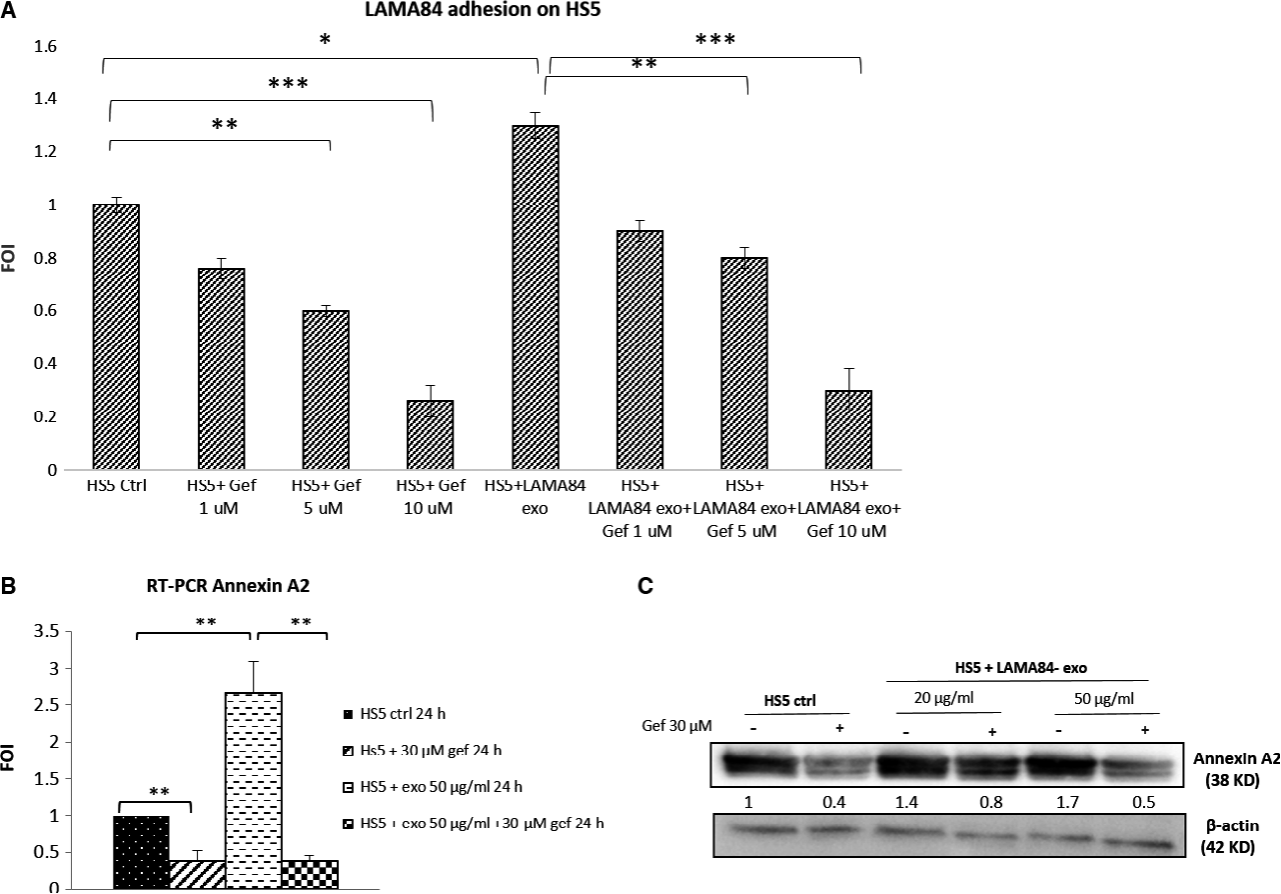
Treatment of HS5 with LAMA84− exosomes increase the LAMA84 cell adhesion to stromal monolayer through the expression of annexin A2. (**A**) For the adhesion assay, HS5 monolayer was treated for 24 h with LAMA84− exosomes (50 μg/ml) ± different doses of gefitinib (1–5–10 μM); after 24 h, LAMA84 cells were added to the different monolayers and were left to adhere for 3.5 h. Pre‐treatment of HS5 monolayer with LAMA84− exosomes promotes the adhesion of chronic myelogenous leukaemia (CML) cells to stromal cells. Gefitinib reverts this effect. Results from three independent experiments are shown. *P < 0.05; **P < 0.01; ***P < 0.001. (**B**) Analysis, by real‐time PCR, of annexin A2 mRNA expression from HS5 treated for 24 h with LAMA84− exosomes (50 μg/ml) ± 30 μM gefitinib. LAMA84− exosomes induce a significative increase in annexin A2 mRNA expression and gefitinib reverts this effect. Levels of the target transcript were normalized to GAPDH as endogenous control. Final values were expressed as fold of induction. Results from three independent experiments are shown. **P < 0.01. (**C**) Total proteins were extracted from HS5 cells and HS5 treated for 24 h with LAMA84− exosomes (20–50 μg/ml) ± 30 μM gefitinib and were subjected to Western blot analysis with antibody against annexin A2. LAMA84− exosomes induce an increase in annexin A2 in a dose‐dependent manner, and co‐treatment of LAMA84− exosomes and gefitinib partially reverts this effect confirming the involvement of epidermal growth factor receptor (EGFR). Numbers represent the values of densitometric analysis of annexin A2 levels, normalized *versus* β‐actin, used as loading control. Results from three independent experiments are shown.

In the bone marrow microenvironment, the bidirectional cell–cell crosstalk between leukaemic and stromal cells is mediated by soluble cytokines and cell–cell adhesion. Figure 6A shows that the pre‐treatment of HS5 for 24 h with LAMA84 exosomes increases the adhesion of LAMA84 cells to stromal monolayer, as previously demonstrated 12. Gefitinib reverts the effect of LAMA84− exosomes treatment in CML cell adhesion to HS5 monolayer, thus indicating the involvement of EGFR pathway in the adhesion of leukaemic cells to stromal cells. Recently, it has been demonstrated that annexin A2, expressed on stromal cells, regulated bone marrow homing of Multiple Myeloma cells supporting their growth and regulating their adhesion to stromal cells 27. To determine whether annexin A2 is involved in our system, we treated HS5 cells with LAMA84 exosomes for 24 h; as shown in Figure 6B, LAMA84 exosomes induce a significative increase in annexin A2 mRNA expression, and data were confirmed at protein level as shown in Figure 6C. Treatment with Gefitinib reverts the effect of LAMA84 exosomes treatment both for annexin A2 mRNA and protein level, suggesting a regulation of annexin A2 by EGFR.

We, therefore, conclude that EGFR plays a critical role in adhesion of leukaemic cells to stromal cells, and we suggest that this adhesion pathway could be mediated by the increase in annexin A2 expression in stromal cells.

## Discussion

In these recent years, the scientific community has focused the attention on the role of the tumour microenvironment and intercellular communication between cancer cells and normal stromal cells. In this context, a number of studies investigated the role played by cancer cell–derived exosomes as cargos of cytokines, growth factors, mRNA and miRNAs in the modulation of tumour progression 7, 8. Exosomes are now considered as signalling organelles that have been demonstrated to affect growth, invasion, angiogenesis and metastatic niche formation 9.

We previously demonstrated that leukaemic and stromal cells establish a bidirectional crosstalk; CML exosomes induce the production and release of IL8 from stromal cells that in turn sustains the growth and survival of CML cells both *in vitro* and *in vivo* 12. The results shown herein suggest a possible mechanism through which CML exosomes cause increased release of IL8 from stromal cells. The EGFR is a signal transducer that has been highly conserved during evolution and plays an important role in different physiological processes, such as cell proliferation, cell migration, angiogenesis and apoptosis.

A number of studies have demonstrated that *in vitro* treatment of mesenchymal stem cells with epidermal growth factor, HBEGF, AREG or TGFα induces growth factor production 28, 29 14 that may also affect malignant phenotypes such as cancer cell proliferation and metastasis 30, 31. Among EGFR ligands, amphiregulin, even if has lower binding affinity for EGFR than EGF or TGFα, is highly expressed in different tumours, such as breast cancer or hepatocellular carcinoma, contributing to the transformed phenotype 17, 18. AREG activates signalling in an autocrine manner in tumour cells, but it is also able to activate EGFR *via* paracrine signals from non‐resident cells of the microenvironment 21. Singh and Coffey recently described a new extracrine (exosomal targeted receptor activation) signalling that involves the packaging and release of EGFR ligands *via* exosomes 19, and Coffey's group demonstrated that tumour exosomes, carrying the EGFR ligand AREG, are rapidly internalized in recipient human breast and colon cancer cells leading to cancer cell invasion 20.

We speculate that a similar mechanism could occur in bone marrow microenvironment and that AREG is involved in the activation of EGFR downstream signalling in mesenchymal stromal cells leading to the expression and release of IL8 and other downstream targets. We have shown that AREG found in LAMA84− exosomes activates EGFR signalling in human stromal cells through a paracrine mechanism and that gefitinib, a specific inhibitor of EGFR activation, or neutralizing antibody anti‐AREG may revert the effects mediated by LAMA84− exosomes. Importantly, exosomes isolated from serum of CML patients carry AREG, thus confirming the role of AREG–EGFR axis mediated by CML exosomes, also *in vivo*.

EGFR downstream signalling, involved in cell–cell communication and tumour progression, is known to lead to the regulation of gene expression through the transcriptional factor SNAIL 25, 26. SNAIL expression has been linked, in several experimental systems, to a change in cell morphology and invasive properties of the cells. Although SNAIL is widely expressed in early development, in adult animals it is limited to a subset of mesenchymal cells, where it has a largely unknown function. Batle *et al*. demonstrated *in vivo* that SNAIL is required to maintain MSCs, and this effect is associated to the responsiveness to TGF‐β1 32.

It is well known that SNAIL induces the expression of different targets, such as MMPs, and directly regulates IL8 expression. Moreover, the SNAIL/IL8 axis plays a critical role in colorectal cancer stemness and malignancy, thus suggesting that IL8 may function as a significant regulatory factor within the tumour microenvironment 33. The data present herein showed that the EGFR signalling, activated in stromal cells by CML exosomes, induces the downstream expression of SNAIL and consequently the increase in SNAIL targets, MMP9 and IL8. We further confirmed, through the use of short‐interfering RNAs, the involvement of SNAIL in CML exosomes‐mediated EGFR signalling in mesenchymal stromal cells.

Our previous work demonstrated that CML exosomes, through the increase in IL8, promote leukaemia cell adhesion on HS5 stromal cells 12. It is well known that EGFR modulates several steps of cancer progression and one of the first steps is adhesion of cancer cells to the non‐tumour cells in the microenvironment. For this reason, we decide to investigate the possible role of EGFR pathway in the adhesion of CML cells to stromal monolayer. We confirmed here that pre‐treatment of HS5 with LAMA84 exosomes increases the adhesion of leukaemic cells to the stromal monolayer; moreover, the treatment with gefitinib reverts these effects, confirming a possible role of EGFR in regulating leukaemic cells adhesion inside the tumour microenvironment of CML.

We investigated the molecules involved in the cell–cell adhesion. Accumulating evidence suggests that annexin A2 is involved in various cellular activities, including cell proliferation, adhesion, migration, invasion and angiogenesis and its overexpression was observed in many types of tumour tissues thus contributing to cancer progression 34, 35. Annexin A2 interacts with several signalling proteins, regulators/effectors of the EGFR/Ras pathway, such as Src tyrosine kinases, Rho, Cdc42, Ras and GTPase activating protein (GAP), thus indirectly modulating EGFR/Ras signalling 36, 37. Grewal and Enrich described annexins as modulators of EGF receptor signalling and trafficking 38. In addition to its expression in cancer cells, annexin A2 is expressed by other cell types found in tumour microenvironment, thus contributing to cancer progression. For example, Shiozawa *et al*. demonstrated that annexin A2, expressed on bone marrow cells, is involved in stem cell homing mechanisms necessary for prostate cancer cells to gain access to the niche 39; recently, it has been shown that annexin A2–CXCL12 interactions regulate metastatic prostate cancer cell targeting and their growth in the bone marrow 40. Furthermore, it has been recently demonstrated that annexin A2, expressed on stromal cells, regulates bone marrow homing of Multiple Myeloma cells supporting their growth, through the activation of ERK ½ and AKT, and regulating their adhesion to stromal cells 23. Interestingly, Seckinger's group demonstrated that annexin A2 expression in primary myeloma cells is an adverse prognostic factor 41 and an increased expression of annexin A2 has been also reported as a poor prognostic factor for solid tumours such as pancreatic and breast cancer 42, 43. We demonstrated that pre‐treatment of HS5 with LAMA84− exosomes increases the expression of annexin A2 at mRNA and protein level and the adhesion of leukaemic cells to the stromal monolayer. Gefitinib reverts these effects, confirming a possible role of EGFR in regulating cancer cell adhesion. Briefly, we have shown here a possible mechanism through which, in the context of the bidirectional crosstalk between bone marrow–derived stromal and leukaemic cells, CML exosomes exert their effect on tumour microenvironment. The involvement of EGFR signalling in this crosstalk and the regulation of growth and survival mediated by Snail and annexin A2 provides a new piece in the understanding of the role of exosomes in this crosstalk and has significant implications for new therapeutic approaches for early diagnosis of leukaemia.

## Conflict of interest

The authors declare that there is no conflict of interest that could be perceived as prejudicing the impartiality of the research reported.

## Author contributions

Conceived and designed the experiments: CC, LS, GDL and RA. Performed the experiments: CC, LS and SR. Analysed the data: CC, LS and RA. Wrote the article: CC, LS and RA. All authors have read and approved the final version of the article.

## Ethics approval

The ethics committee of the University Hospital P. Giaccone approved this study. The committee's reference number is 10/2012.

## Supporting information


**Data S1** Supplementary Materials and Methods.

**Figure S1** LAMA84− exosomes treatment increases EGFR phosphorylation in HS5 stromal cells.

**Figure S2** Expression of EGFR ligands in CML patient cells and LAMA84 cells.

**Figure S3** LAMA84− exosomes increase IL8, Snail and MMP9 expression in bone marrow primary stromal cells.
