# Supplementary material for: Chronic myelogenous leukaemia exosomes modulate bone marrow microenvironment through activation of epidermal growth factor receptor
Source: J Cell Mol Med. 2016 May 14;20(10):1829–39. doi: 10.1111/jcmm.12873 (PMC4876029; doi:10.1111/jcmm.12873)
Supplement: Supplementary file 1 — Data S1 Supplementary Materials and Methods. [file JCMM-20-1829-s001.docx]

**Supplementary Materials and Methods**

**RTK Assay**

HS5 cells were starved for 2h in serum free medium and treated or not for different times (30’, 6h, 24h) with 50 μg/ml of LAMA84- exosomes. Subsequently the cells were lysed with specific lysis buffer provided with the kit and subjected to Human Phospho RTK Array, according to the manifacturer’s protocol (R&D Systems, Abingdon, UK).
