# Supplementary figures and images for: Chronic myelogenous leukaemia exosomes modulate bone marrow microenvironment through activation of epidermal growth factor receptor
Source: J Cell Mol Med. 2016 May 14;20(10):1829–39. doi: 10.1111/jcmm.12873 (PMC4876029; doi:10.1111/jcmm.12873)

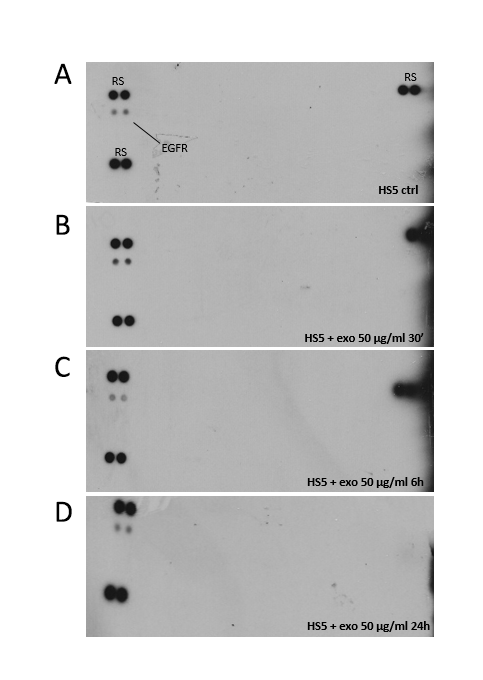

Supplement: Supplementary file 2 — Figure S1 LAMA84− exosomes treatment increases EGFR phosphorylation in HS5 stromal cells. [file JCMM-20-1829-s002.tif]

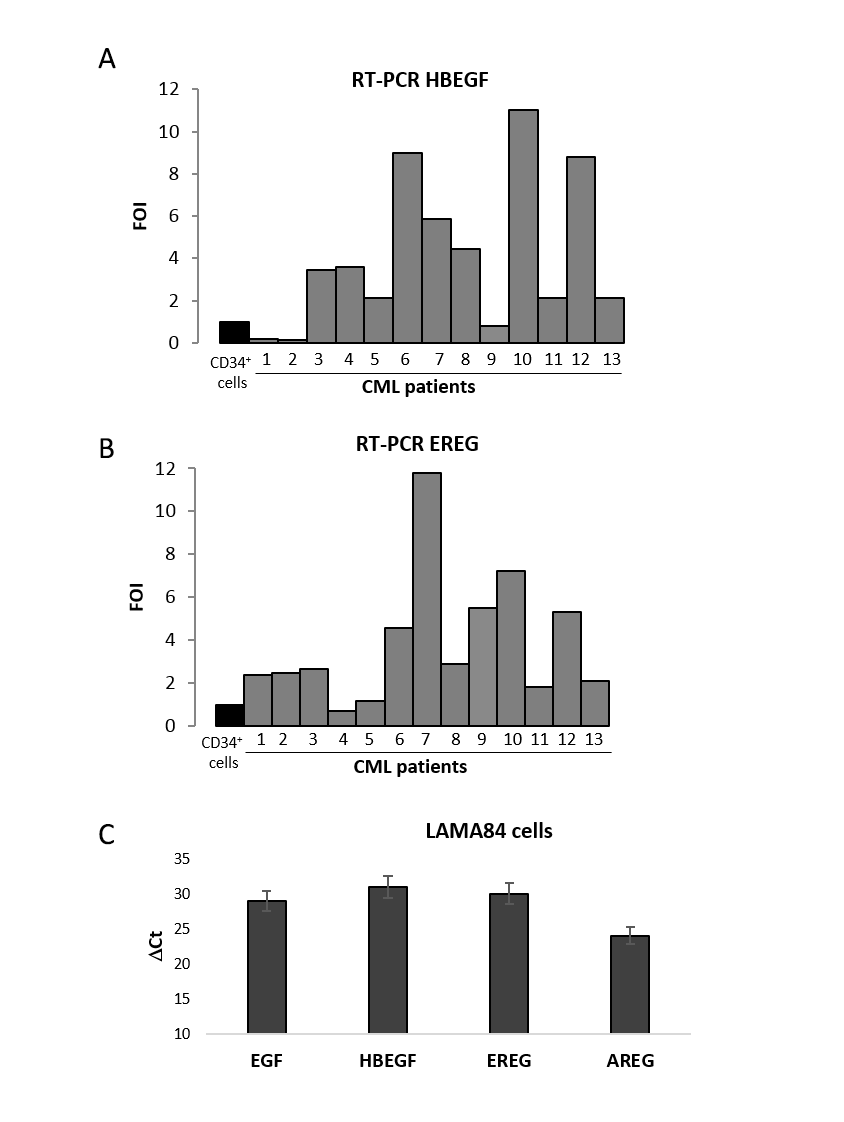

Supplement: Supplementary file 3 — Figure S2 Expression of EGFR ligands in CML patient cells and LAMA84 cells. [file JCMM-20-1829-s003.tif]

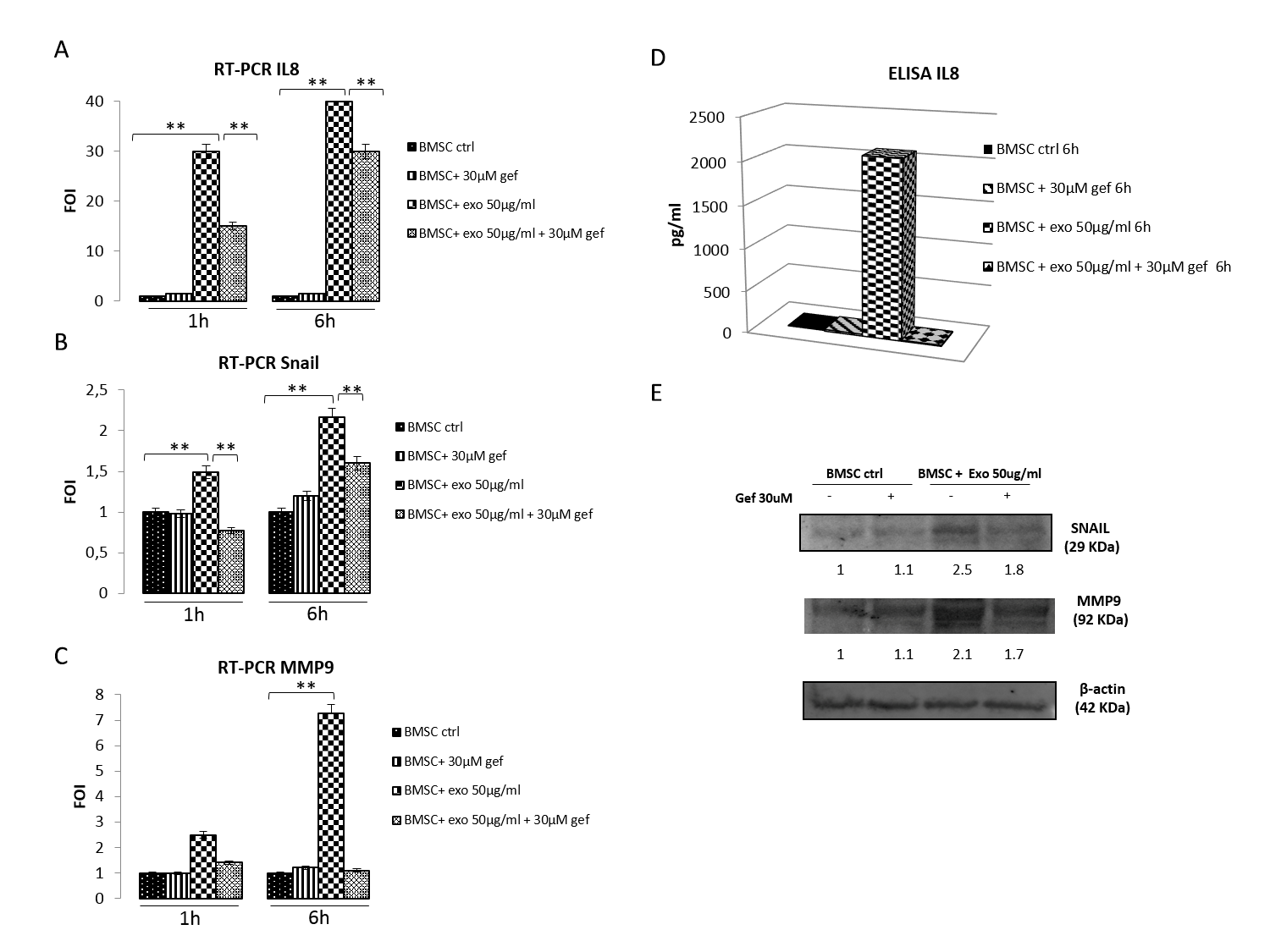

Supplement: Supplementary file 4 — Figure S3 LAMA84− exosomes increase IL8, Snail and MMP9 expression in bone marrow primary stromal cells. [file JCMM-20-1829-s004.tif]
